# Prediction of Residual Compressive Strength after Impact Based on Acoustic Emission Characteristic Parameters

**DOI:** 10.3390/polym16131780

**Published:** 2024-06-24

**Authors:** Jingyu Zhao, Zaoyang Guo, Qihui Lyu, Ben Wang

**Affiliations:** 1 The System Design Institute of Mechanical-Electrical Engineering, Beijing 100854, China; jingyuu_zhao@163.com; 2 School of Science, Harbin Institute of Technology, Shenzhen 518055, China; 3 Research Institute of Interdisciplinary Science, School of Materials Science and Engineering, Dongguan University of Technology, Dongguan 523808, China

**Keywords:** low-velocity impact, residual compressive strength, acoustic emission, machine learning

## Abstract

This study proposes a prediction method for residual compressive strength after impact based on the extreme gradient boosting model, focusing on composite laminates as the studied material system. Acoustic emission tests were conducted under controlled temperature and humidity conditions to collect characteristic parameters, establishing a mapping relationship between these parameters and residual compressive strength under small sample conditions. The model accurately predicted the residual compressive strength of the laminates after impact, with the coefficient of determination and root mean square error for the test set being 0.9910 and 2.9174, respectively. A comparison of the performance of the artificial neural network model and the extreme gradient boosting model shows that, in the case of small data volumes, the extreme gradient boosting model exhibits superior accuracy and robustness compared to the artificial neural network. Furthermore, the sensitivity of acoustic emission characteristic parameters is analyzed using the SHAP method, revealing that indicators such as peak amplitude, ring count, energy, and peak frequency significantly impact the prediction results of residual compressive strength. The machine-learning-based method for assessing the damage tolerance of composite laminates proposed in this paper utilizes the global monitoring advantages of acoustic emission technology to rapidly predict the residual compressive strength after the impact of composite laminates, providing a theoretical approach for online structural health monitoring of composite laminates. This method is applicable to various composite laminate structures under different impact conditions, demonstrating its broad applicability and reliability.

## 1. Introduction

Carbon-fiber-reinforced polymer (CFRP) composites have been widely used in aerospace, automotive and marine fields due to their advantages in structural efficiency and performance [1,2,3,4]. However, due to the weak interfacial properties of CFRP laminated composites, they are prone to internal delamination, debonding, and other hidden damages when facing low-velocity impacts (LVI) such as tool drops, hailstorms, and bird strikes [5,6]. Such damages can significantly reduce the out-of-plane and bending properties of composite structures, making them one of the most common types of damage in composite structures, which severely affects their structural integrity and safety [7,8,9]. Therefore, it is necessary to evaluate the damage tolerance of composite structures under low-velocity impact loads.

At present, the prediction of compression strength after impact on composite laminates mainly relies on experiments and numerical simulations, with both of which it is difficult to meet the demand for rapid evaluation of structural residual strength in terms of efficiency and cost [10,11]. Therefore, it is necessary to carry out predictions of the residual strength of CFRP composite laminates during their service life so as to make timely assessments of structural reliability. In recent years, the rise of machine learning methods has provided new insights for the assessment of damage tolerance, mainly focusing on neural network algorithms [12,13]. Neural networks are utilized to establish mapping relationships between impact parameters (such as impact energy, impact angle, etc.) and residual compression strength [14,15]. However, the application of neural networks is limited by their demand for large amounts of data and the lack of experience in designing network structures.

Acoustic emission (AE) technology is a powerful non-destructive testing method that is widely used for real-time monitoring of damage in materials. AE refers to the phenomenon where transient elastic waves are generated by the rapid release of energy from localized sources within a material, typically due to crack formation and propagation. This technology is particularly suitable for detecting and evaluating damage in composite materials, as it can capture dynamic events related to internal structural changes. AE sensors are attached to the material’s surface to detect these elastic waves, and the data collected provide valuable insights into the type, location, and severity of the damage.

This article selected carbon-fiber-reinforced composite materials as the research object and conducted low-speed impact tests under eight different energies (10–45 J). During the impact process, acoustic emission technology was utilized to assess the damage degree of the laminate, and the characteristic parameters of the acoustic emission data collected during the impact were extracted. Subsequently, the damaged laminate was subjected to compression tests after impact to obtain the residual compressive strength of the post-impact structure. Due to the insufficient data, an extreme gradient boosting model was adopted to establish the correspondence between the acoustic emission characteristic parameters and residual compressive strength under different impact energies. A comparative analysis was conducted with an artificial neural network. Additionally, the SHAP method was utilized to analyze the sensitivity of the acoustic emission characteristic parameters, revealing that the peak amplitude, ring count, energy, and peak frequency had significant impacts on the prediction results of the residual compressive strength. The purpose of this study is to assess the health status of composite material structures within their actual service life, thereby reducing maintenance time and providing new insights for online structural health monitoring and intelligent sensing of composite materials.

## 2. Experiments and Methods

### 2.1. Materials and Specimens

**Figure 1:**
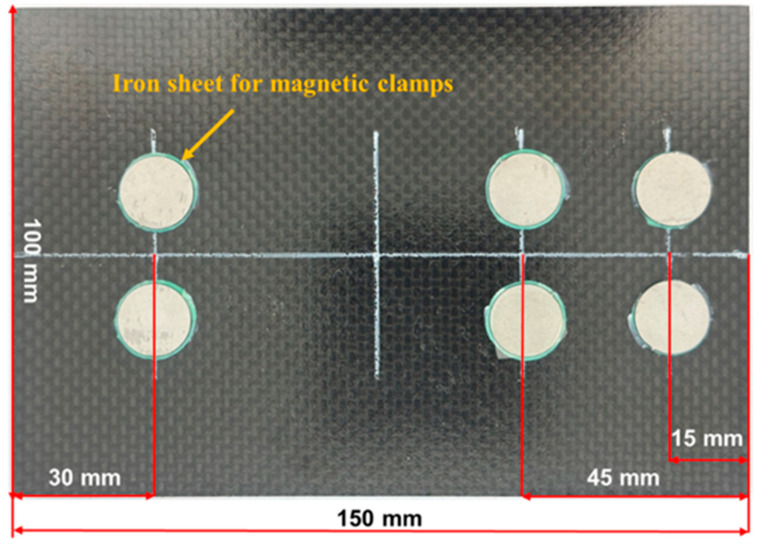
Low-velocity impact laminate specimens.

The CFRP composite laminates were stacked by 20 layers of carbon fiber plain weave prepreg with a layering angle of 0°. Each layer of the prepreg is composed of T300 carbon fiber fabric and epoxy resin (with a resin volume fraction of approximately 42%), and has a thickness of approximately 0.25 mm. The laminates were cut into standard test specimens with dimensions of 150 mm × 100 mm, and the average thickness of the tested specimens was 5.1 ± 0.10 mm. To fix the magnetic clamp for the acoustic emission sensor, a circular iron sheet was bonded to the specimen using an adhesive film. The curing temperature for the adhesive film was 120–140℃, and the curing time was 2–3 h. The dimensions of the specimen and the bonding position of the patch are shown in Figure 1, where the diameter of the patch is 15 mm and the thickness is 1 mm.

### 2.2. Low-Velocity Impact Test and Result Analysis

**Figure 2:**
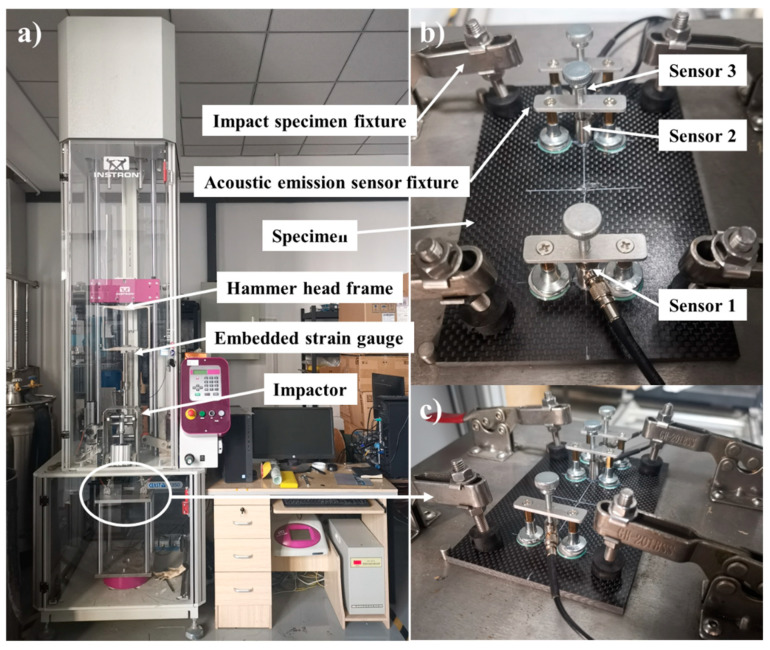
The low-velocity impact experimental setup: (**a**) CEAST 9350 drop tower impact tester; (**b**) acoustic emission sensor installation method; (**c**) the low-velocity impact test fixture.

According to the ASTM D7136/D7136M standard [16], the low-velocity impact tests were conducted on the CFRP composite laminates using the drop-weight impact system, Instron CEAST 9350, manufactured by Instron CEAST, a company under the ITW Group, which originated from the Norwood, MA, USA. The test setup is illustrated in Figure 2. A steel hemispherical impactor with a diameter of 16 mm is selected, with a total mass of 5.5 kg, including the counterweight. By adjusting the preset height of the impactor, different impact energies can be obtained. Figure 2 shows the low-velocity impact test platform, where the installation method of the acoustic emission sensor is as depicted in Figure 2b. The magnetic clamp can be attached to the iron sheet, pressing the acoustic emission sensor against the upper surface of the composite laminate. The impact samples were divided into eight groups based on different impact energies, specifically 10 J, 15 J, 20 J, 25 J, 30 J, 35 J, 40 J, and 45 J. To ensure the reliability and repeatability of the test results, three tests were conducted under each impact energy.

**Figure 3:**
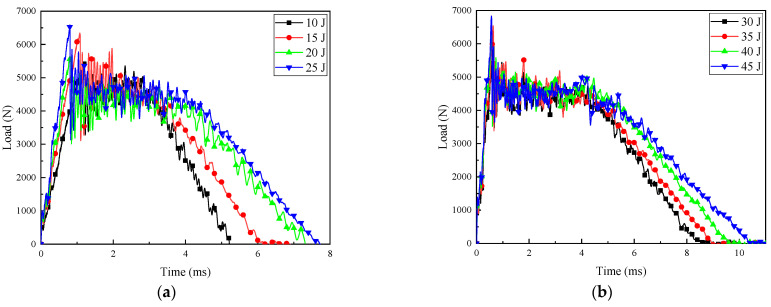
Load–time curves of composite laminates at different impact energies: (**a**) 10–25 J; (**b**) 30–45 J.

**Figure 4:**
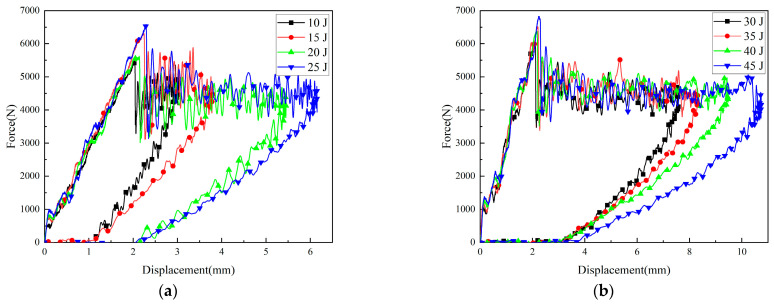
Force-displacement curves of composite laminates at different impact energies: (**a**) 10–25 J; (**b**) 30–45 J.

The force–time curves and load–displacement curves of CFRP laminates under different impact energies are shown in Figure 3 and Figure 4, respectively. A slight decrease in load could be observed at the initial stage of all load–time curves, which was known as Hertz failure force ($F_{h}$), indicating the beginning of minor damage. After reaching the maximum value, the load curves under all impact energies showed a certain degree of sharp decrease, followed by a period of relatively stable multiple oscillations. Finally, a decreasing trend was observed again. This indicated that under the impact of energy, significant changes occurred in the energy conversion and release process of the CFRP laminates. The sudden drop in load denoted that obvious damage behaviors such as cracking, delamination, and fracture had occurred in the resin and fibers, leading to a decrease in the stiffness of the laminates [17]. Therefore, even if only a small stress is applied, the sample can still produce a relatively large strain.

Within the impact energy range of 10 J to 25 J, as the impact energy increased, the loading time required to reach the peak load gradually decreased. At the same time, the loading phase of the load was approximately linear, and the slope increased with the increase in impact energy. Within the impact energy range of 30 J to 45 J, the load slopes for different energy levels were basically consistent during the linear stage. It is worth noting that the load undergoes multiple oscillations after reaching its maximum value until the loading is complete. This is due to the inherent frequency caused by the loosely connected components of the impactor, as well as the influence of bending vibrations in the impacted specimen [18].

### 2.3. Compression after Impact Test and Result Analysis

**Figure 5:**
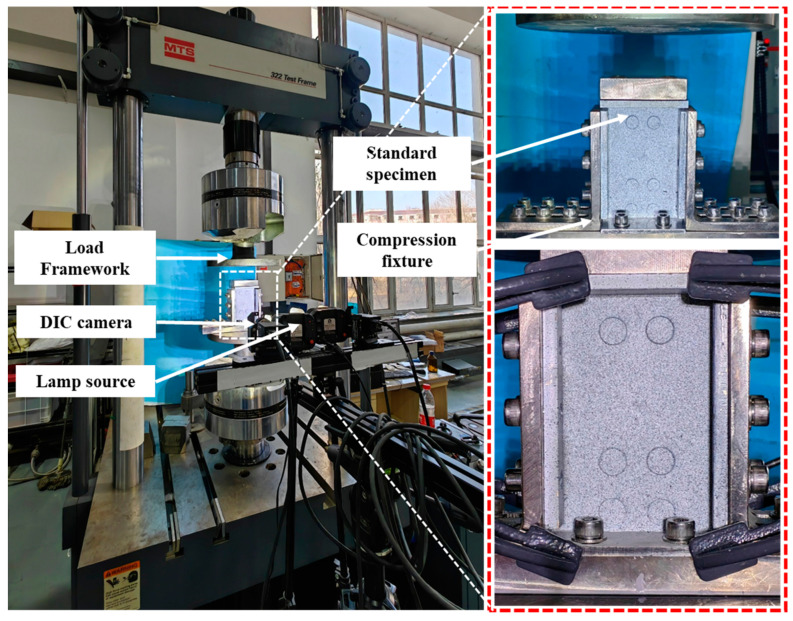
Compression after impact test platform.

According to the ASTM D7137M-17 standard [19], a compression test is conducted on the CFRP composite laminate after impact. The test apparatus employed was the STM 322 testing machine, as shown in Figure 5. The compression test was conducted using displacement control, with a loading speed of 0.5 mm/min. The three-dimensional digital image correlation (DIC) method was used to measure and monitor the displacement and strain on the surface of the specimen during the compression test after impact, using the Correlated Solutions VIC-3D equipment. Digital Image Correlation (DIC) is an optical method that utilizes image registration techniques to accurately measure two-dimensional and three-dimensional changes in images. This method is commonly used for non-contact, full-field displacement and strain measurements [20].

**Figure 6:**
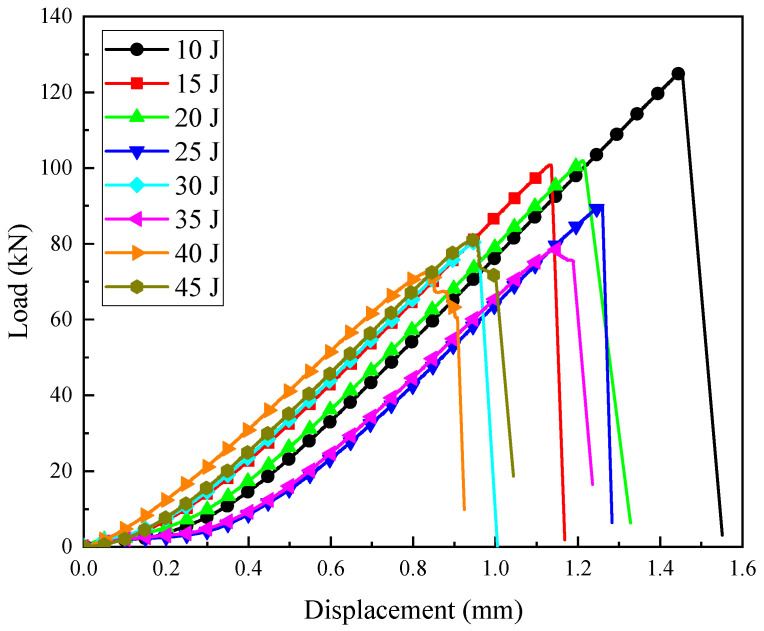
CAI test load–displacement curves under different impact energies.

The load–displacement curves of the specimens under different impact energies are shown in Figure 6. These results were from typical individual tests and are meant to illustrate specific instances of specimen behavior under impact conditions. The trend of the compression load–displacement curve of the damaged laminates under various impact energies was the same. Each curve had a maximum value, which was the maximum load that the laminate could bear. After reaching the extreme point, the load rapidly decreased, indicating that the test specimen had completely failed and could not bear any further compression load. The observations showed that during the initial stage of loading, there was a short section of the load–displacement curve where the slope remained almost unchanged, with a range of approximately 0–0.3 mm. Subsequently, the load–displacement curve rose gently and then its slope value became larger and remained unchanged until the test specimen finally failed and was invalid. The reason for this phenomenon is that it is difficult to fully contact the indenter with the test piece, and the test piece with the fixture base during the initial stage of compressive load loading, resulting in a smaller nominal stiffness of the test piece in the early loading stage.

**Figure 7:**
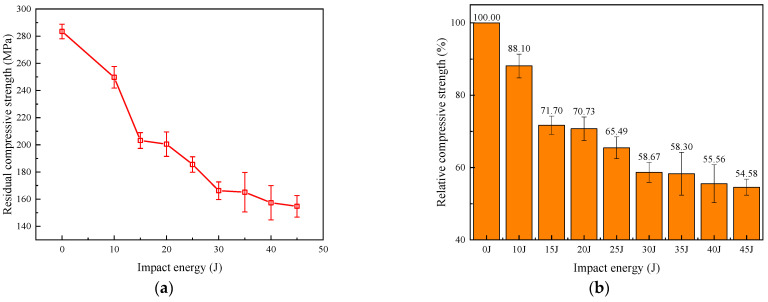
CAI strength under different impact energies: (**a**) absolute value; (**b**) relative value.

Figure 7 presents averaged residual compressive strength results derived from multiple tests at each impact energy level. Averaging the data helps to account for variability and provides a more reliable representation of the general trends and overall performance across all tested specimens. As shown in Figure 7a, the residual compressive strength of the laminate under different impact energies gradually decreases as the impact energy increases. As the impact energy increased, severe deformation and damage occurred in the matrix and fibers, resulting in a decrease in the mechanical properties of the material. When the impact energy was relatively small, the material could mitigate the impact by absorbing and dispersing the energy. However, when the impact energy was large, the material was unable to effectively absorb and disperse the energy, leading to extensive damage and failure within the material. These damages and failures could lead to a decrease in the strength and stiffness of the material, subsequently reducing its residual compressive strength after impact. Therefore, as the impact energy increased, the residual compressive strength of the composite material after impact decreased accordingly [21].

To more intuitively observe the degree of strength reduction, the relative value of residual strength was calculated, defined as the ratio of the maximum compressive strength of the damaged specimen to the strength of the undamaged specimen. The results are shown in Figure 7b. With the increase in impact energy, the damage to the specimen gradually expanded, resulting in a rapid decrease in residual compressive strength. When the impact energy was 15 J, the compressive strength of the specimen was approximately 71.70%. Subsequently, the residual compressive strength decrease curve tended to flatten. This is because delamination damage was the main reason for the decrease in residual compressive strength [22]. At higher impact energies, the delamination area only extended slightly, and the impact energy was primarily dissipated through fiber breakage.

## 3. Prediction of Residual Compressive Strength

**Figure 8:**
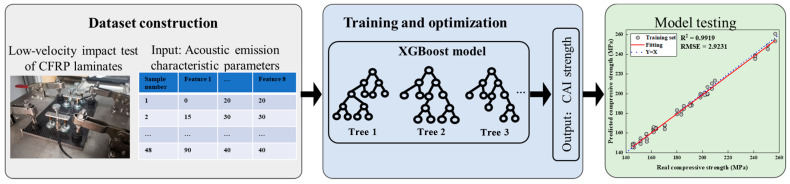
Flow chart of CAI strength prediction based on acoustic emission characteristic parameters.

This section proposed a method for predicting the post-impact compressive residual strength of composite laminates based on acoustic emission characteristic parameters. The flowchart, as shown in Figure 8, is divided into three main components: dataset construction, training and optimization of the machine learning model, and model testing.

In the dataset construction phase, we collected acoustic emission (AE) data from carbon-fiber-reinforced polymer (CFRP) composite samples subjected to low-velocity impacts. The AE signals were recorded using high-sensitivity sensors during the impact tests conducted under controlled conditions. The characteristic parameters of the AE signals, such as peak amplitude, ring count, energy, and peak frequency, were extracted from the waveforms. These parameters were then labeled with the corresponding residual compressive strength values obtained from subsequent compression tests after impact. For the training and optimization of the machine learning model, we employed an extreme gradient boosting (XGBoost) algorithm due to its robustness and efficiency with small datasets. The training process involved feeding the training data into the model and adjusting the hyperparameters to minimize the prediction error. Hyperparameters such as the learning rate, maximum depth of trees, and number of estimators were optimized using a grid search method in combination with cross-validation to prevent overfitting and ensure the model’s generalizability. Once the model was trained and optimized, it was tested on the unseen test dataset to evaluate its predictive performance. The test set was used to assess the model’s ability to generalize to new data.

### 3.1. Construction of Characteristic Parameter Dataset

**Figure 9:**
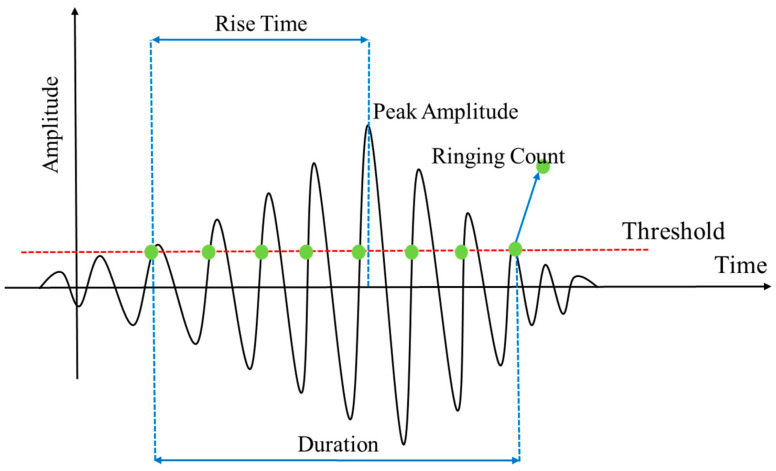
Definition of acoustic emission characteristic parameters.

First, common acoustic emission signal characteristic parameters are defined. A typical acoustic emission waveform is shown in Figure 9, and the corresponding characteristic parameters are defined as follows [23].

1. Peak amplitude: the maximum voltage of the signal, measured in volts (V) or decibels (dB).
2. Duration: the time interval between the first and last threshold crossings, measured in microseconds (µs).
3. Rise time: the time interval between the first threshold and the maximum amplitude, measured in microseconds (µs).
4. Ringing count: the number of times the waveform crosses the threshold in the increasing direction during the duration of the waveform.
5. Energy: the area under the squared waveform during its duration, measured in mV·mS.
6. RMS voltage: The root mean square value of the signal level over the sampling time, measured in mV.
7. Average signal level: the average value of the signal level during the sampling time, measured in millivolts (mV).
8. Peak frequency: the frequency corresponding to the highest amplitude in the frequency distribution obtained from the fast Fourier transform of the signal, measured in kilohertz (kHz).

Eight commonly used characteristic parameters were extracted from the acoustic emission signal waveforms of the composite laminates during the impact process as input data for the machine learning model. These parameters included peak amplitude, duration, rise time, ringing count, energy, RMS voltage, average signal level, and peak frequency. The residual compressive strength of the laminates measured in Section 2.2 was used as the output data for the machine learning model. 

The main parameter settings for the acoustic emission equipment were as follows: trigger threshold of 10 dB, peak definition time (PDT) of 50 µs, hit definition time (HDT) of 150 µs, and hit lockout time (HLT) of 300 µs. There were eight groups of impact energies, with three tests conducted for each energy level. Each impact test generated three samples from three sensors, resulting in a total of 72 sample groups. The dataset was randomly divided into a training set and a test set with a split ratio of 75% to 25%.

### 3.2. Residual Strength Prediction Based on XGBoost Model

**Figure 10:**
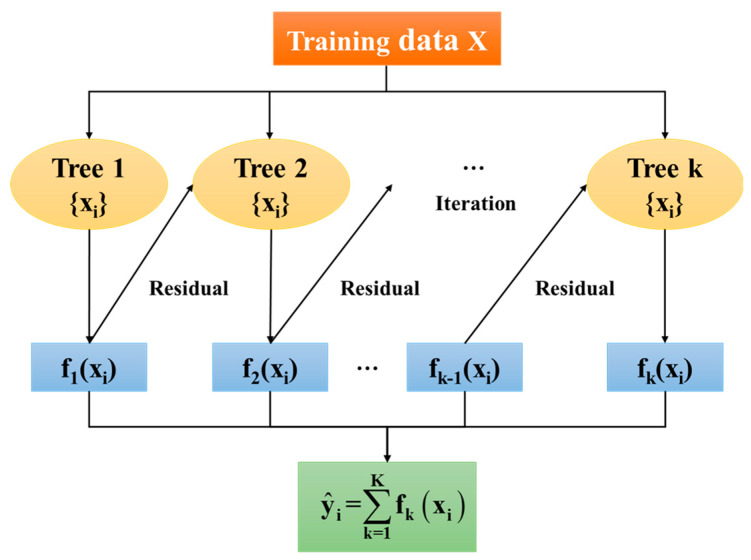
Schematic diagram of extreme gradient boosting model structure.

Extreme gradient boosting (XGBoost) is an algorithm based on decision trees that belongs to the category of supervised machine learning methods [24]. XGBoost belongs to the category of ensemble machine learning methods. Its core idea is to combine multiple weak learners (decision trees) to create a stronger model. The XGBoost model utilizes the gradient boosting algorithm to systematically improve the performance of the model by optimizing a differentiable loss function. Compared to other machine learning algorithms, the XGBoost model is capable of flexibly modeling based on different types of data. The XGBoost model employs various regularization techniques, which can help prevent overfitting and improve generalization performance [25]. This model has been widely applied in fields such as meteorology, environmental science, and engineering [26,27,28,29]. Figure 10 illustrates a typical structure of the XGBoost model, which optimizes the loss function by iteratively adding decision trees to the model. Each tree attempts to correct the errors of the previous tree, and the parameters of each tree are updated based on the residuals of the previous tree. The final prediction is the weighted sum of the predictions from all trees.

The derivation process of the extreme gradient boosting model is as follows [24]:

For a dataset $D=\left\{\left(x_{i},y_{i}\right)\right\}\left(|D|=n,x_{i}\in R^{m},y_{i}\in R\right)$ with n samples and m features for each sample, the ensemble model based on regression trees uses *K* functions to predict the output as follows:

(1) $${\hat{y}}_{i}=\phi \left(x_{i}\right)=\sum_{k=1}^{K}f_{k}\left(x_{i}\right),f_{k}\in ℱ$$

And $x_{i}$ represents the vector of input samples, K is the number of trees, f is the scoring function of a single decision tree, and $ℱ=\left\{f(x)=w_{q(x)}\right\}\left(q:R^{m}\to T,w\in R^{T}\right)$ corresponds to the function space composed of all regression trees, which is the set of all possible regression trees.

Each scoring function $f_{k}$ corresponds to an independent tree structure q and leaf weights w. Each regression tree contains a continuous weight on each leaf, with $w_{i}$ representing the weight on the ith leaf. For a given sample, it is classified into the corresponding leaf node through the decision rules in the tree (given by q), and the final prediction is calculated by summing the weights in the corresponding leaf nodes (given by w). To learn the set of functions used in the model, the following regularized loss function is minimized:

(2) $$ℒ(\phi )=\sum_{i}l\left({\hat{y}}_{i},y_{i}\right)+\sum_{k}\Omega \left(f_{k}\right)$$

And $\Omega (f)=\gamma T+\frac{1}{2}\lambda \|w{\|}^{2}$. l is the loss function, which measures the difference between the predicted value ${\hat{y}}_{i}$ and the true value $y_{i}$. Ω is the regularization term that controls the complexity of the model, *T* is the number of leaves, and γ and λ are the control parameters for the complexity and scaling factor of each leaf, respectively.

The ensemble model in Equation (2) includes functions as model parameters, making it impossible to optimize using traditional optimization methods in Euclidean space. Assuming that ${{\hat{y}}_{i}}^{\left(t\right)}$ is the predicted value for the i-th sample at the t-th iteration, it is necessary to add *f* to minimize the following loss function:

(3) $${ℒ}^{\left(t\right)}=\sum_{i=1}^{n}l\left(y_{i},{{\hat{y}}_{i}}^{\left(t-1\right)}+f_{t}\left(x_{i}\right)\right)+\Omega \left(f_{t}\right)$$

Performing a second-order Taylor expansion on it yields:

(4) $${ℒ}^{\left(t\right)}≃\sum_{i=1}^{n}\left[l\left(y_{i},{\hat{y}}^{\left(t-1\right)}\right)+g_{i}f_{t}\left(x_{i}\right)+\frac{1}{2}h_{i}f_{t}^{2}\left(x_{i}\right)\right]+\Omega \left(f_{t}\right)$$

$g_{i}=\partial_{{\hat{y}}^{\left(t-1\right)}}l\left(y_{i},{\hat{y}}^{\left(t-1\right)}\right)$ and $h_{i}=\partial_{{\hat{y}}^{\left(t-1\right)}}^{2}l\left(y_{i},{\hat{y}}^{\left(t-1\right)}\right)$ are the first-order and second-order gradient statistics of the loss function, respectively. After removing the constant term and defining $I_{j}=\left\{i|q\left(x_{i}\right)=j\right\}$ as the set of samples in leaf j, the simplified loss function for the t-th step is obtained.

(5) $$\begin{array}{cc} {\tilde{ℒ}}^{\left(t\right)} & =\sum_{i=1}^{n}\left[g_{i}f_{t}\left(x_{i}\right)+\frac{1}{2}h_{i}f_{t}^{2}\left(x_{i}\right)\right]+\gamma T+\frac{1}{2}\lambda \sum_{j=1}^{T}w_{j}^{2}\\ & =\sum_{j=1}^{T}\left[\left(\sum_{i\in I_{j}}g_{i}\right)w_{j}+\frac{1}{2}\left(\sum_{i\in I_{j}}h_{i}+\lambda \right)w_{j}^{2}\right]+\gamma T \end{array}$$

The scoring function that measures the quality of the tree structure q is as follows:

(6) $${\tilde{ℒ}}^{\left(t\right)}(q)=-\frac{1}{2}\sum_{j=1}^{T}\frac{{\left(\sum_{i\in I_{j}}g_{i}\right)}^{2}}{\sum_{i\in I_{j}}h_{i}+\lambda }+\gamma T$$

Generally, enumerating all possible tree structures can be challenging. Therefore, to simplify this process, a greedy approach is adopted. The splitting process starts with a single leaf node, calculates the gain after splitting, and iteratively adds more branches to the tree gradually. Since the random selection of training data can affect model accuracy, 10-fold cross-validation was used to train the extreme gradient boosting model. The training set was divided into 10 equal parts, and in each iteration, nine parts were used to train the model while the remaining part was used for testing. The model was evaluated using the average of the results from the 10 iterations.

**Figure 11:**
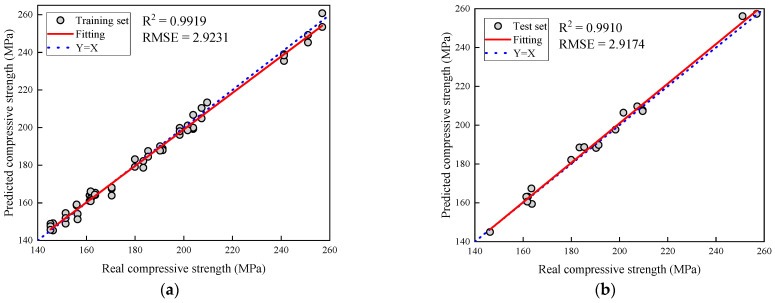
Comparison of predicted and real values of remaining compressive strength after impact: (**a**) training set; (**b**) test set.

Figure 11 illustrates the predicted residual compressive strength of CFRP composite laminate under various impact energies, alongside their actual values. It is noteworthy that the R^2^ values obtained from the extreme gradient boosting model for both the test and training sets remained consistent, indicating minimal overfitting. The predictive model demonstrates the ability to accurately forecast the residual compressive strength of the composite laminate post-impact, within a studied range of approximately 140–260 MPa. The coefficient of determination (R^2^) on the test set is 0.9910, with a root mean square error (RMSE) of 2.9174. The proximity of R^2^ to 1 signifies the model’s high accuracy and reliable prediction of residual compressive strength. This outcome validates the superior performance of the extreme gradient boosting model utilized in handling small data volumes and showcases robustness in practical applications.

**Figure 12:**
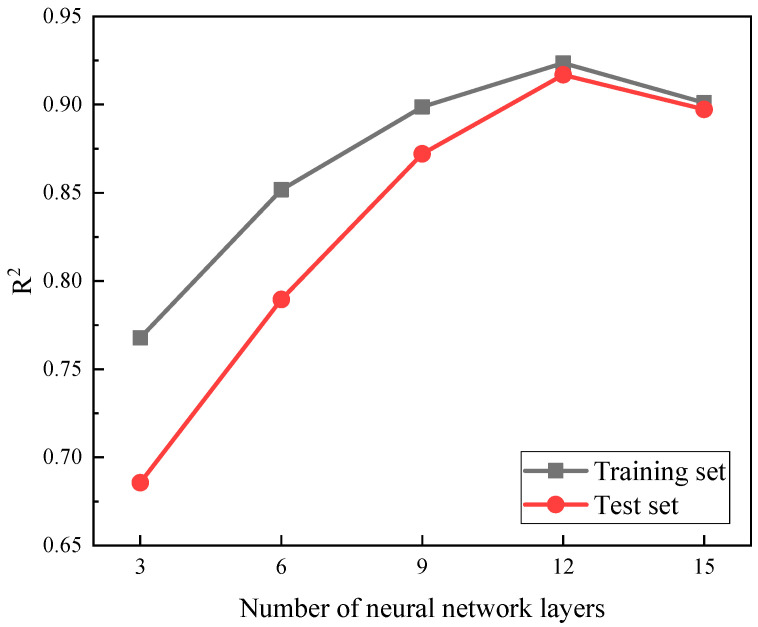
Comparison results of decision coefficients of artificial neural network models with different numbers of layers.

In addition, to further validate the effectiveness of the XGBoost model, this study compared its prediction results with those of the Artificial Neural Networks (ANN) model in terms of residual compressive strength. Five different layer numbers, namely 3, 6, 9, 12, and 15, were selected to construct artificial neural networks, and the impact of different neural network structures on prediction performance was compared. In each layer, the number of neurons was set to 15. The comparison results of the coefficient of determination for different network structures with varying layer numbers are shown in Figure 12. When the number of neural network layers was set to three, the coefficient of determination for the training set and test set of the model were 0.7677 and 0.6856, respectively. The significantly lower coefficient of determination for the test set compared to the training set indicated the occurrence of a notable overfitting phenomenon in the model. As the number of network layers increased, the overfitting phenomenon was alleviated to a certain extent. The model achieved the best fit to the data when the number of layers reached 12. This was attributed to the fact that with the increase in the number of layers, the model had more parameters involved in training, thereby enhancing its ability to learn the nonlinear mapping relationship between acoustic emission characteristic parameters and residual compressive strength. Notably, when the number of layers increased to 15, the performance of the model slightly decreased. This was due to the current amount of data being insufficient to fully train the parameters of a 15-layer artificial neural network, resulting in an underfitting phenomenon in the model.

**Figure 13:**
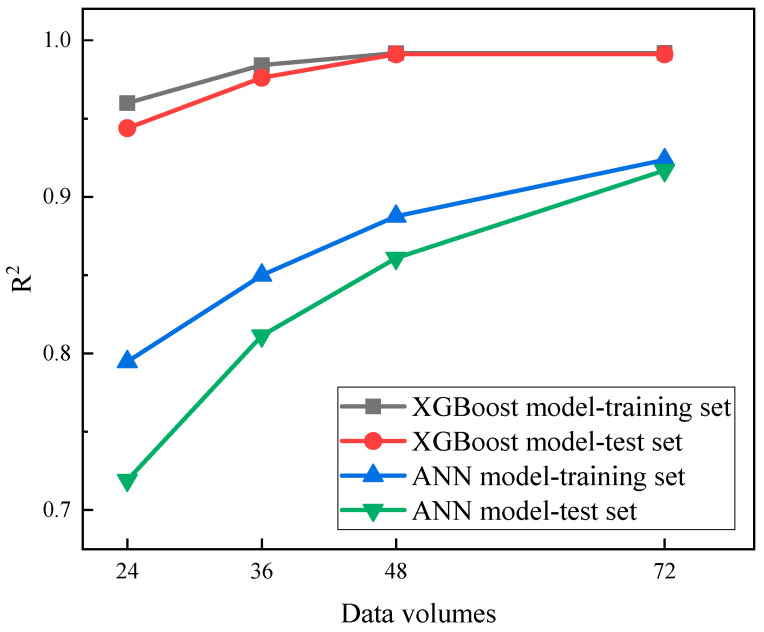
Comparison of the coefficient between XGBoost model and ANN model with different data volumes.

Subsequently, a comparison was conducted to assess the impact of varying data volumes on the predictive performance of both the extreme gradient boosting model and the artificial neural network model. For the latter, the structure with the optimal performance, consisting of 12 hidden layers, was employed. Figure 13 illustrates the coefficient of determination (R^2^) for both models across different data volumes. It is evident that under conditions of limited data, the XGBoost model maintained superior predictive performance, with a significantly higher R^2^ compared to the artificial neural network model. Additionally, the minimal difference in R^2^ between the training and test sets denoted that the model did not exhibit overfitting. Evidently, compared to artificial neural networks, the XGBoost model demonstrated superior generalization capabilities and resistance to overfitting, exhibiting comparable predictive performance on both the test set and the training set. Additionally, it exhibited relatively strong adaptability to small sample sizes, enabling the achievement of satisfactory predictive performance even with limited data.

### 3.3. Sensitivity Analysis of Characteristic Parameters

In this section, the sensitivity analysis of acoustic emission characteristic parameters is conducted using the SHapley Additive ExPlanations (SHAP) method [30]. The SHAP method is an approach used to interpret the prediction results of machine learning models. It is based on the theory of Shapley values and aims to explain the contribution of each feature to the prediction outcome. The core idea of utilizing the SHAP method to calculate feature sensitivity is that the greater the absolute value of the Shapley value, the more important the feature is.

**Figure 14:**
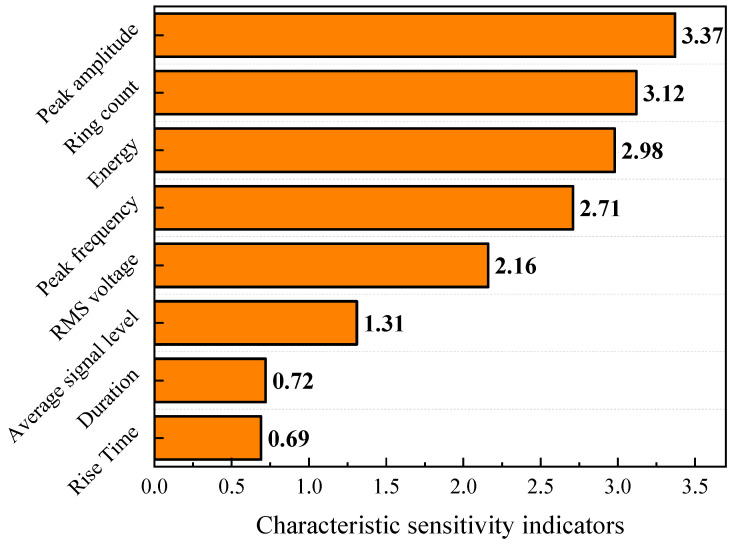
Sensitivity ranking of acoustic emission characteristic parameters.

Based on the distribution of average SHAP values, sensitive factors that significantly influence the prediction results of residual compressive strength can be identified among the acoustic emission characteristic parameters, as shown in Figure 14. The peak amplitude had the greatest impact on the prediction results of residual compressive strength, with a sensitivity index reaching 3.37. The ring count, energy, and peak frequency exhibited an influence on the prediction of residual compressive strength that was second only to the peak amplitude. The duration and rise time have the lowest impact on the prediction results of residual compressive strength. As the impact energy increased, large-scale damage occurred in the matrix and fibers within the composite material, which manifested in the form of acoustic emission characteristic parameters such as amplitude and ring count. Consequently, these acoustic emission characteristic parameters were highly correlated with the damage of the composite material, and the more severe the damage, the lower the residual compressive strength of the composite material.

## 4. Conclusions

This paper utilized acoustic emission data obtained during the impact process of CFRP laminated composite plates to predict their residual compressive strength after impact. The main conclusions are as follows:

Impact tests and residual compressive strength experiments were conducted on CFRP laminates under different impact energies. The results revealed that the impact resistance and residual compressive strength of the specimens decreased as the impact energy increased. And the rate of decline in residual compressive strength decelerated with higher impact energy. This trend can be attributed to the fact that delamination damage is the primary cause of reduced compressive strength. However, at higher energy levels, delamination zones expand marginally, and the impact energy is primarily dissipated through fiber fractures, resulting in a lower rate of strength degradation.

A prediction method for residual compressive strength after impact based on the extreme gradient boosting model was developed and compared with the performance of the artificial neural network model. The results show that, compared with artificial neural networks, the limit gradient enhancement model exhibits superior accuracy and robustness with a coefficient of determination (R^2^) of 0.9910 and a root mean square error (RMSE) of 2.9174 on the test set when the data volume is 72. Furthermore, the sensitivity analysis of acoustic emission characteristic parameters using the SHAP method revealed that factors such as peak amplitude, ring count, energy, and peak frequency had a significant impact on the prediction results of residual compressive strength.

## Data Availability

The raw/processed data required to reproduce these findings cannot be shared at this time as the data also form part of an ongoing study.
